# Quality of Dabai Pulp Oil Extracted by Supercritical Carbon Dioxide and Supplementation in Hypercholesterolemic Rat—A New Alternative Fat

**DOI:** 10.3390/foods10020262

**Published:** 2021-01-27

**Authors:** Noor Atiqah Aizan Abdul Kadir, Azrina Azlan, Faridah Abas, Intan Safinar Ismail

**Affiliations:** 1 Department of Nutrition, Faculty of Medicine and Health Sciences, Universiti Putra Malaysia, Serdang 43400 UPM, Selangor, Malaysia; atiqahaizan@yahoo.com; 2 Research Centre for Excellence for Nutrition and Non-Communicable Disease, Faculty of Medicine and Health Sciences, Universiti Putra Malaysia, Serdang 43400 UPM, Selangor, Malaysia; 3 Halal Products Research Institute, Universiti Putra Malaysia, Serdang 43400 UPM, Selangor, Malaysia; 4 Department of Food Science, Faculty of Food Science and Technology, Universiti Putra Malaysia, Serdang 43400 UPM, Selangor, Malaysia; faridah_abas@upm.edu.my; 5 Department of Chemistry, Faculty of Science, Universiti Putra Malaysia, Serdang 43400 UPM, Selangor, Malaysia; safinar@upm.edu.my

**Keywords:** alternative fat, antioxidant, anti-inflammatory, dabai pulp oil, hypercholesterolemia

## Abstract

Dabai pulp oil (DPO) is new oil extracted from the pulp of *Canarium odontophyllum*. The quality and efficacy of DPO are needed to promote its potential as a new alternative fat. Therefore, we investigate the quality of DPO, which includes moisture and volatile content (MVC), free fatty acid content (FFA), iodine value (IV), and peroxide value (PV). Furthermore, we evaluate the efficacy of DPO against hypercholesterolemia elicited by a high-cholesterol diet in rats. The MVC of DPO was <0.001 ± 0.00%. Next, the FFA in DPO was 2.57 ± 0.03%, and the IV of DPO was 53.74 ± 0.08 g iodine/100 g oil. Meanwhile, the PV of DPO was 4.97 ± 0.00 mEq/kg. Supplementation of DPO in hypercholesterolemic rats for 30 days revealed the hypocholesterolemic effect (significant reduction of total cholesterol, triglyceride, and 3-hydroxy-3-methylglutaryl-CoA reductase) accompanied by a significant reduction of inflammatory markers (C-reactive protein, interleukin-6 and tumour necrosis factor-α), and lipid peroxidation (MDA). We also observed a significant improvement of lipoprotein lipase (LPL) and antioxidant capacities (total antioxidant status, superoxide dismutase, glutathione peroxidase, and catalase) of the rats. The results on the quality and efficacy of locally made DPO suggest its potential use as a healthy alternative fat in the future.

## 1. Introduction

*Canarium odontophyllum* or “dabai” is an exotic seasonal fruit in Sarawak, Malaysia. This fruit grows in abundance, especially in the Sibu and Kapit regions of Sarawak, [1]. Dabai pulp oil (DPO) is a new vegetable oil source extracted from the pulp of *Canarium odontophyllum.* The potential of dabai fruit to be exploited as a new fat source was first initiated using the conventional solvent extraction technique [2,3]. Oleic (18:1), linoleic (18:2 cis n-6), and palmitic (16:0) acids are the most abundant fatty acids in the oil. The fatty acid composition of DPO is similar with palm oil, characterised by a near equal percentage of saturated fatty acids (SFA) and monounsaturated fatty acids (MUFA), at about 40% each, and polyunsaturated fatty acids (PUFA) at about 12–13% [2,3].

Furthermore, New Zealand white rabbits fed with a diet containing 2% of DPO showed significant improvement in their lipid profile (increased plasma HDL-cholesterol and reduced plasma LDL-cholesterol and triglyceride levels), as well as a reduced TBARS (thiobarbituric acid reactive substances) level. The noticeable effect in levels of plasma malonaldehyde, superoxide dismutase, glutathione peroxidase, and total anti-oxidant status in healthy rabbits suggests the potential of DPO as a new alternative vegetable oil [4]. Although the previous solvent extracted DPO findings showed promising effects in New Zealand white rabbits, the conventional chloroform-methanol extraction could be toxic due to solvent residue in the extracted oil. 

Extraction of oil using supercritical fluid (SFE) is considered a novel technique applied for oil processing. The characteristics of the final product are easily altered by changing the process parameters [5]. Earlier, we had performed supercritical carbon dioxide (SC-CO_2_) extraction at 40 °C and 40 MPa as an alternative technique to produce a non-toxic DPO. The identified fatty acids in DPO were SFA (47.65 ± 0.11%), MUFA (40.38 ± 0.79%), and PUFA (13.11 ± 1.10%). Interestingly, the extracted oil showed a similar trend of near-equal percentage of SFA and MUFA at about 40% each, and PUFA at about 13%. The main component of DPO was palmitic acid (41.56 ± 0.10%), followed by oleic acid (39.37 ± 1.01%) and linoleic (cis) acid (12.54 ± 1.03%). DPO is an SFA-rich oil due to its high SFA composition (47.65 ± 0.11%) [6,7].

The semi-polar coumaric acid and anisic acid are the primary phenolic acids in DPO. Additionally, prenyl-naringenin, sophoraflavanone B, and neobavaisolavone are the flavonoid compounds identified in DPO [8]. Further, we had evaluated the safety of using SC-CO_2_ extracted DPO by acute toxicity study in Specific Pathogen Free Sprague-Dawley rats. At a single dose of 5000 mg/kg, the extract did not cause any acute toxicity effects or mortality to rats’ treatment during observation periods in 14 days [7].

In Malaysia, palm oil is the most extensively used edible oil. Malaysian used palm oil, due to its benefits such as competitive price, superior frying quality and oxidative stability owing to the high content of MUFA and SFA compared to other edible oils such as corn oil, sunflower oil, and olive oil [9]. The investigation of DPO indirectly adds variety to the types of vegetable oil for commercialisation. In quality control, an inspection of the chemical properties such as moisture and volatile content, free fatty acid, iodine value, and peroxide value are critical as these determine the shelf-life quality and economic value of oils [10].

Production of DPO by SFE is still relatively new, and there are no published data available regarding the quality parameter of SC-CO_2_ extracted DPO in Malaysia. To the best of our knowledge, this is the first study to examine the moisture, volatile content, free fatty acid, iodine value, and peroxide value in SC-CO_2_ extracted DPO. This work aims to evaluate the quality parameters of SC-CO_2_ extracted DPO and the efficacy of DPO against hypercholesterolemia elicited by a high-cholesterol diet in rats, to promote the potential of DPO as a new source of vegetable oil or solid fats.

## 2. Materials and Methods

### 2.1. Supercritical Carbon Dioxide Extraction of Dabai Pulp Oil (DPO)

**Figure 1:**
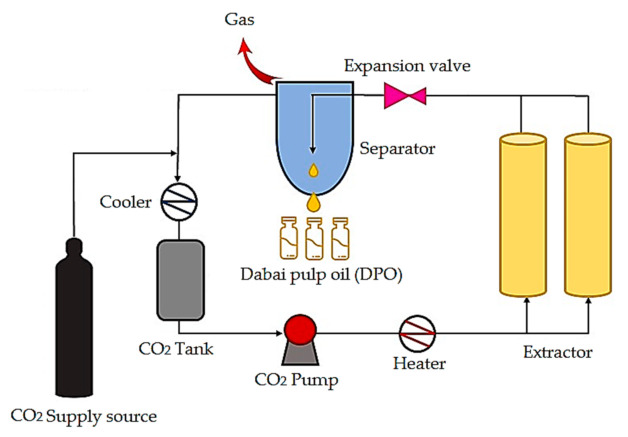
Schematic diagram of the supercritical carbon dioxide extraction system.

We collected fresh dabai fruits (226 kg) from Sarikei Sarawak, Malaysia. The identification and preparation of dabai pulp were performed based on the method described in our previous study [11]. Freeze-dried dabai pulp (48.92 kg) was subjected to SC-CO_2_ extraction. The extraction condition for DPO was performed at a pressure of 40 MPa with a temperature of 40 °C. The extraction protocol was executed as described in the method from our previous study [6]. DPO was stored in the chiller (4 °C) until further use. The SC-CO_2_ extraction system is illustrated in Figure 1.

### 2.2. Quality Evaluation of DPO

#### 2.2.1. Determination of Moisture and Volatile Content

The determination of moisture and volatile content in the DPO was carried out using the Malaysian Palm Oil Board (MPOB) test method: MPOB p2.1:2004 [12]. A petri dish was heated in an oven at 103 ± 2 °C to eliminate the moisture and volatile substances. Next, 10 g of DPO sample was weighed into a petri dish and later cooled in the desiccators. The petri dish with the DPO sample was placed in an oven at 103 °C for 2.5 h. Later the sample was cooled at room temperature (23–25 °C) in a desiccator for 30 min, followed by weighing. This step was repeated until a constant weight was achieved. The moisture and volatile content were calculated by:

MVC (%) = [(*M_b_* − *M_d_*)/(*M_b_* − *M*)] × 100 (1)

where, *M* = mass (g) of the dish, *M_b_* = mass (g) of the dish and oil, and *M_d_* = mass (g) of the dish and oil after drying.

#### 2.2.2. Determination of Free Fatty Acid

The determination of free fatty acid in the DPO was conducted using the MPOB test methods, p2.5:2004 [12]. The free fatty acid content in DPO was expressed as the percentage of oleic acid. The DPO sample was weighed into a conical flask. Next, neutralised isopropanol (50 mL) was added to the flask, followed by a few phenolphthalein drops. Later, the conical flask was placed on a hot plate at 40 °C. The flask was gently swirled at the same time as being titrated with sodium hydroxide (NaOH) standard solution until the appearance of the first permanent pink colour. The free fatty acid content was expressed as:

FFA as oleic acid (%) = (28.2 × *M* × *V*)/*m* (2)

where, 28.2 = equivalent factors for oleic acid, *M* = molarity (mol L^–1^) of standard NaOH solution, *V* = volume (mL) of the standard NaOH solution used and *m* = mass (g) of the oil sample.

#### 2.2.3. Determination of Iodine Value

The measurement of the iodine value in DPO was carried out by using MPOB test methods, p3.2:2004 [12]. A DPO sample (0.2 g) was mixed with 20 mL mixture of cyclohexane and glacial acetic acid. Later the mixture was reacted with 25 mL of Wijs reagent. Next, 20 mL of 100 g/L potassium iodide and 150 mL distilled water were added to the mixture after storage in the dark for 1 h. The solution was then titrated with 0.1 M sodium thiosulfate until the yellowish iodine colour disappeared. A small amount of starch solution was put into the solution as an indicator. The titration continued until the blue colour also disappeared. The following equation calculates the iodine value:

IV (g iodine/100 g oil) = [12.69*C* (*V*_1_ − *V*_2_)]/*m* (3)

where 12.69 = transfer equivalent thiosulphate to g (iodine), *C* = concentration of sodium thiosulfate solution (mole L^−1^), *V*_1_ = volume (mL) of sodium thiosulfate solution used for the blank test, *V*_2_ is the volume (mL) of sodium thiosulfate solution used for the sample, and *m* = is the mass (g) of the sample.

#### 2.2.4. Determination of Peroxide Value

The determination of peroxide value was carried out by using AOAC Official Method 965.33 [13]. DPO (5 g) was placed into 250 mL of a conical flask and then mixed and swirled to dissolve with 30 mL of acetic acid and chloroform solvent mixture (3:2). Next, 1 mL of potassium iodide solution was added to the solution. The solution was allowed to stand for 1 min in a dark room with occasional shaking, and then 30 mL of distilled water was added. Later, the solution was titrated with 0.01 N sodium thiosulphate solution until the yellow colour was gone. Next, 1 mL of starch solution indicator was added. The titration was continued with vigorous shaking to release all I_2_ from CH_3_Cl layer until the blue colour disappeared. The peroxide value was estimated using the following equation:

PV (mEq/kg) = (*V* × *N* × 100)/*W* (4)

where, *V* = volume of sodium thiosulphate, *N* = normality of sodium thiosulphate solution, and *W* = weight of the sample.

### 2.3. Animal Experiment

Male-specific pathogen-free (SPF) Sprague-Dawley rats (n = 24) at the age of 4 weeks, weighing between 100 to 150 g were purchased from Nomura Siam International Co., Ltd. (Bangkok, Thailand), Thailand. All the animals were acclimatised for two weeks under individual ventilated cages (IVC) at the Comparative Medicine and Technology Unit (COMeT) Universiti Putra Malaysia with a controlled temperature between 21 to 23 °C, at relative humidity in a range of 50 to 60% with regular light and dark cycles and free access to food and water. During housing, animals were monitored twice daily for health status and general toxicity signs under veterinarian supervision. No adverse events were observed throughout the experiments. The animal housing, experiments and procedures, and animals’ deposition were located at the Comparative Medicine and Technology Unit (COMeT) Universiti Putra Malaysia. All experiment protocols and ethical aspects were carefully followed and performed following the proper use and care of laboratory animals, as approved by the Institutional Animal Care and Use Committee (IACUC), Universiti Putra Malaysia (IACUC R045/2015).

The animals were randomised into control rats (NG; n = 6), which received normal diet (ND), whereas, the remaining rats (n = 18) received a high cholesterol diet added with 1% cholesterol (HC). After 30 days, all experimental rats fasted overnight. Later, the rats were intraperitoneally anaesthetised with ketamine (50 mg/kg body weight) and xylazine (10 mg/kg body weight) by a veterinarian. Blood (1 mL) was collected via cardiac puncture for hypercholesterolemia screening. The blood was withdrawn slowly to prevent the heart from collapsing. Rats with total serum cholesterol and LDL-C significantly higher than NG rats were considered hypercholesterolemia rats [14]. After confirming the hypercholesterolemia model’s establishment, the rats fed with the high cholesterol diet were randomised into three groups (n = 6) and subjected to different dietary regimens for another 30 days of the treatment period. The main characteristics of the experimental groups and dietary regimens during the treatment period are as follows:

1. Control group (NG), animals received a normal diet.
2. The hypercholesterolemic positive control group (PG), animals received a high cholesterol diet added with 1% cholesterol.
3. Hypercholesterolemic 2% DPO-treated group (HG), animals received a high cholesterol diet added with 1% cholesterol and 2% DPO.
4. Hypercholesterolemic simvastatin treated group (SG), animals received a high cholesterol diet added with 1% cholesterol. Simvastatin (10 mg/kg/day) was administered orally to rats.

At the end of the treatment period (day 60), rats were intraperitoneally anaesthetised with ketamine (50 mg/kg body weight) and xylazine (10 mg/kg body weight) by a veterinarian. Approximately 3 mL of blood was collected into sterile tubes via cardiac puncture; with prior euthanisation by exsanguination via a cardiac puncture through the heart’s abdominal aorta.

### 2.4. Experimental Diet

The normal diet (without added cholesterol) was prepared by properly mixing the correct amounts of corn starch, sucrose, casein, cellulose, mineral mixture, vitamin mixture, DL-methionine, choline, corn oil, and ghee. The mixture was stirred thoroughly, spread on baking pans, cut into smaller pieces, and baked in an oven (Binder ED23, Tuttlingen, Germany) at 50–60 °C for 24 h. Similarly, the high cholesterol diets were prepared using the same method as the cholesterol-free diet with 1% cholesterol added [15]. For the DPO treatment diet, 2% of DPO was incorporated into the diets to meet the required diet regimens (Table 1). All rats were given 25 g of the respective experimental diets daily. All diets were stored at 4 °C, and fresh pallets were provided daily to the rats.

### 2.5. Efficacy Evaluation of DPO

Blood collected in a sterile tube was centrifuged at 3000 rpm at 4 °C for 10 min. The serum was stored at −80 °C until the day of measurement. The biochemical tests were performed using Dimension^®^ Xpand^®^ Plus (Siemens Healthcare Diagnostics, Newark, DE, USA) according to the manufacturer’s instructions for each parameter, total cholesterol (TC) (Siemens Healthcare, DF27), triglyceride (TG) (Siemens Healthcare, DF69A), low-density lipoprotein (LDL) (Siemens Healthcare, DF131) and high-density lipoprotein (HDL) (Siemens Healthcare, DF48B). The 3-hydroxy-3-methylglutaryl-CoA reductase (HMG-CoA-r) and lipoprotein lipase (LPL) were analysed by using the rat HMG-CoA ELISA kit and the rat LPL ELISA kit, respectively (Wuhan Fine Biotech Co., Ltd., Wuhan, China). Total anti-oxidant status (TAS) was determined using a rat TAS ELISA kit (Hangzhou Sunlong Biotech Co., Ltd., Hangzhou, China). Superoxide dismutase (SOD) was analysed by using the rat SOD ELISA kit. Meanwhile, catalase (CAT) and glutathione peroxide (GPx) were measured by using the rat CAT assay kit and the rat GPx assay kit, respectively (Elabscience Biotechnology Inc., Wuhan, China). The level of lipid peroxidation (MDA) was determined using the rat MDA ELISA kit (Wuhan Fine Biotech Co., Ltd., Wuhan, China). The C-reactive protein (CRP) level was measured using the rat CRP ELISA kit (Elabscience Biotechnology Inc., Wuhan, China). Whereas, interleukin-6 (IL-6) and tumour necrosis factor-α (α-TNF) were quantified by using the rat IL-6 ELISA kit and rat α-TNF ELISA kit, respectively, (eBioscience, San Diego, CA, USA). All procedures were conducted attentively and precisely according to the manufacturer’s instructions.

### 2.6. Statistical Analysis

Data were expressed as the mean ± standard deviation (n = 6). Data were analysed by using one-way ANOVA using SPSS for Windows version 23. Duncan’s multiple range test was used to test whether there were significant differences among the experimental groups. Values are considered statistically significant when *p* < 0.05.

## 3. Results and Discussion

### 3.1. The Quality of DPO

Under this study’s conditions, the apparent yield of DPO from SC-CO_2_ extraction was 18.07% (mass of extract/mass of dry matter). The extraction yield (mass of extract/mass of dry matter) was applied to indicate the extraction conditions’ effects [16]. The extraction yield of DPO in this study was lower than SC-CO_2_ extracted palm kernel oil (46.9%) [17] as well as canola oil (33.61%) [18]. Many factors affect the oil yield from the SFE method, including pressure, temperature, moisture content of the raw material, particle size, flow rate, processing time, and co-solvent [19]. For example, the previously mentioned canola oil [18] was obtained through SC-CO_2_ extraction with ethanol as a co-solvent. A combination of co-solvents in the SFE system led to improved yield and selectivity of the products and a reduced fractionation step [20]. Meanwhile, the palm kernel oil [17] was extracted through SC-CO_2_ extraction via combined pressure swing extraction. The yield was about double the yield of continuous extraction for a given pressure and extraction time. When applying combined pressure swing, the increased yield noted is due to the hold-up of carbon dioxide in the extractor. This situation allows the carbon dioxide to penetrate the pericarp structure of palm kernel oil. Hence, when high pressure was applied, the oil’s solubility increased due to an increase in the carbon dioxide density [21].

The quality parameters of DPO, which include moisture and volatile content (MVC), free fatty acid (FFA), iodine value (IV), and peroxide value (PV) were determined to demonstrate the quality and stability of DPO. Table 2 shows the MVC (<0.001 ± 0.00%), FFA (2.57 ± 0.03%), IV (53.74 ± 0.08), and PV (4.97 ± 0.00 mEq/kg) of DPO. Since DPO is the new oil, there is no specifications value developed to inspect the physical and chemical properties of DPO in Malaysia. Our research is the first to document the quality parameter of SC-CO_2_ extracted DPO to the best of our knowledge.

DPO comprised mostly of the SFA (47.65 ± 0.11%), followed by 40.38 ± 0.79% of MUFA and 13.11 ± 1.10% of PUFA [7] (Table 3) (Appendix A Table A1). Based on the fatty acid content (FAC) of DPO, the oil is categorised into the SFA class due to higher SFA content. A similar trend was also seen in chloroform-methanol (CM) extracted DPO (43.42 ± 0.05% SFA, 42.53 ± 0.06% MUFA and 14.05 ± 0.09%, PUFA) [3] and petroleum-ether (PE) extracted DPO (44.3 ± 0.07% SFA, 42.82 ± 0.06% MUFA and 12.76 ± 0.03% PUFA) [2]. The SFA of SC-CO_2_ extracted DPO was higher than CM and PE extracted DPO, yet it has lower MUFA than the CM and PE extracted DPO. Though the PUFA in SC-CO_2_ extracted DPO was higher than the PE extracted DPO, it was lower than the CM extracted DPO.

The main component of DPO was palmitic acid (41.56 ± 0.10%), followed by oleic acid (39.37 ± 1.01%) and linoleic (cis) acid (12.54 ± 1.03%). Palmitic acid in SC-CO_2_ extracted DPO was higher than CM and PE extracted DPO (40.31 ± 0.01% and 36.05 ± 0.05%, respectively). Meanwhile, oleic acid in SC-CO_2_ extracted DPO was lower than CM and PE extracted DPO (41.54 ± 0.06% and 41.9 ± 0.02%, respectively) [2,3]. Although the linoleic (cis) acid in SC-CO_2_ extracted DPO was higher than that of the PE extracted DPO (11.75 ± 0.02%) [3], it was lower compared to CM extracted DPO (14.05 ± 1.96%) [2]. A similar trend was also seen in SC-CO_2_ extracted palm oil (40 °C, 41.4 MPa); the palmitic acid was higher in SC-CO_2_ extracted palm oil (9.8%) than Soxhlet extracted (SE) palm oil (7.5%). Lower oleic acid and linoleic (cis) acid was noted in SC-CO_2_ extracted palm oil (11.9% and 0.9%, respectively) compared to SE extracted palm oil (15.1% and 2.7%, respectively) [22].

It was reported that with lower pressure (20.7–27.6 MPa), a lower amount of shorter chain (C8 C14) was extracted. Meanwhile, at higher pressure (34.5–48.3 MPa), higher long-chain fatty acid constituents (C16–C18:2) were extracted [22]. The higher pressure of SC-CO_2_ was used in this study, explaining a higher percentage of palmitic acid (C16). However, it can be seen that oleic acid (C18:1) and linoleic (cis) acid (18:2 cis n-6) were lower in SC-CO_2_ extracted DPO when compared to CM extracted DPO. This phenomenon is caused by lower solubility as the carbon number increases [23]. Ragunath et al. [24] reported that the solubility of fractionated fatty oil constituents of C6, C12, C16, and C18:1 by using SC-CO_2_ (40–80 °C, 30 MPa) decreased with an increase in carbon number.

However, the ease of operating conditions of the SFE process leads to the prospect of utilising fractionation via SC-CO_2_ extraction [25]. Zaidul et al. [26] demonstrated that SC-CO_2_ could be applied to fractionate palm kernel oil with lower C8–C14 levels and higher C16–C18:2 fatty acid constituents. Hence, fractionation of DPO based on its fatty acid composition plays a significant role in producing DPO with varying physical or nutritional properties of interest to the food industry.

DPO can be grouped into a palmitic acid subclass, as the main component of DPO was palmitic acid. The best example of a palmitic acid subclass is palm oil. The FAC of DPO was comparable to palm oil as DPO is characterised by a near equal percentage of unsaturated fatty acid (UFA) and SFA, based on its fatty acid composition; 39.37% of oleic acid, 41.56% of palmitic acid and 4.31% of stearic acid. The FAC of DPO was within the observed range of FAC for palm oil as specified by the Malaysian Standard MS814:2007 for oleic acid (37.4–44.1%), palmitic acid (39.2–45.8%) and stearic acid (3.7–5.4%) [27]. Due to this exciting data, it was our great interest to compare the quality parameters of DPO with the specification developed by the Malaysian Standard MS814:2007 for crude palm oil (CPO) [27] (Table 2). We also compared the PV of DPO with PV established by Food and Drug Administration (FDA)/World Health Organisation (WHO) under the Codex Alimentarius Commission (1999) for Edible Fats and Oils Not Covered by Individual Standards [28], to strengthen our claim that DPO is safe for human consumption.

Water is one of the most common impurities found in oil products. High moisture content will influence the hydrolytic stability of oil products [29]. Accumulation of water at the bottom of the storage tank can influence the MVC content in oil [30]. Hence, it is essential to control the moisture content within the permissible limit. In this study, the MVC content in DPO (<0.001 ± 0.00%) was lower than the specification moisture content in CPO (0.25%). Based on the result, it is confirmed that DPO is stable under the storage condition (4 °C). Lower MVC content in DPO was attributed to lower accumulation of water at the bottom of the bottle.

Meanwhile, FFA content is an important indicator that determines the quality of oil products as it indicates the economic value of edible oils [29]. During storage, FFAs are released as a result of the hydrolysis reaction of oil. A high FFA content level means that the oil product is rancid and has low quality [31]. It is important to note that FFA content in DPO (2.57 ± 0.03%) in the present study was below the specified FFA for crude palm oil (CPO) (5.0%). The quality of raw material used in the processing of oils and fats and storage condition is an important parameter affecting the FFA value in oils and fats [32]. In this study, dabai fruits were freshly harvested, which took a half day for the process to complete and was then shipped to the Faculty of Medicine and Health Sciences, UPM on the same day of fruit collection. Once the fruits arrived at the facility, the fruits were immediately stored at low temperature (4 °C) before sample preparation. Then, fruits were immediately processed on the next day to avoid deterioration. Deteriorated fruits would yield high FFA in oil and fats [33].

Additionally, DPO was stored in an opaque white plastic bottle. The characteristics of the storage bottle (opaque) reduced light absorption by the stored palm oil, hence decreased the possibility of high FFA and rancidity [33]. Therefore, in this study, DPO was preserved from rancidity.

In addition to FFA, IV content also reflects the oil sample’s vulnerability to oxidation [34]. Several factors contribute to IV oil content: first, a higher IV value is proportional to the UFA in the oil. The previous study showed that the IV content in sunflower oil was 121.00 ± 0.38 g iodine/100 g oil [10]. The higher IV content was a result of higher UFA. Sunflower oil contains high levels of PUFA, which make them susceptible to rancidity [35]. Second, the oxidation of oil was faster at a higher temperature, which will lead to higher IV content. Appropriate storage condition can help prevent the oils from an oxidation and hydrolysis reaction [29]. The IV content of DPO (53.74 ± 0.08 g iodine/100 g oil) in the present study was within the allowable range set for CPO (50.1–54.9 g iodine/100 g oil), and the PUFA in DPO was 13.11 ± 1.10%. Lower IV content for DPO was a result of lower PUFA. Additionally, DPO was stored in a chiller (4 °C) after the oil was extracted. Hence, it can be concluded that the quality of DPO was preserved during the storage period.

PV is an index to quantify the formation of hydroperoxide. The formation of hydroperoxide must be suppressed to protect against oxidation and the secondary oxidised products. This critical process assures the excellent quality of oil that is safe for consumption [36]. The PV of DPO (4.97 ± 0.00 mEq/kg) was higher than the limit of specification for CPO (2.0 mEq/kg); however, the PV of DPO in this present study was lower than the PV of DPO reported by Azlan et al. [2] (7.99 ± 0.01 mEq/kg). Moreover, the PV in DPO was less than 15 mEq peroxide/kg oil, which is well below the level established by Food and Drug Administration (FDA)/World Health Organisation (WHO) under the Codex Alimentarius Commission (1999) [28]. Our data specifies that the DPO in this study has good stability towards oxidation and is considered safe for human consumption.

### 3.2. The Efficacy of DPO in Hypercholesterolemic Rats

In Table 4, hypercholesterolemic rats showed significant elevation of TC, LDL, HMG-CoA-r, MDA, and inflammatory markers (CRP, IL-6, and α-TNF) and significant reduction of the anti-oxidant profile (TAS, SOD, GPx, CAT) when compared to normal rats (*p* < 0.05). These results showed that hypercholesterolemia had been established in the rats using a high cholesterol diet (1% of cholesterol). Additionally, the elevated inflammatory markers and reduced anti-oxidant enzymes in hypercholesterolemic rats indicate that hypercholesterolemia was associated with oxidative stress and inflammation. The difference in lipid profile, inflammatory markers and anti-oxidant enzymes between normal rats and hypercholesterolemic rats fed with high cholesterol diet was demonstrated in previous studies [37,38,39].

The most used drugs for treating lipid disorders are statins [40]. In this study, rats treated with simvastatin had significantly reduced TC, LDL, and HMG-CoA-r compared with hypercholesterolemic rats (*p* < 0.05). Treatment with simvastatin demonstrated a significant increase of SOD and CAT and a significant decrease in MDA compared with hypercholesterolemic rats (*p* < 0.05). Moreover, rats treated with simvastatin also showed significantly lower inflammatory markers (CRP, IL-6, and α-TNF) when compared to hypercholesterolemic rats (*p* < 0.05). Simvastatin is a well-accepted and potent hypolipidemic drug with a known mechanism of action as an HMG-CoA reductase inhibitor in cholesterol biosynthesis [41]. Further to lipid-lowering, simvastatin reduces the circulating MDA, CRP and pro-inflammatory cytokines, including IL-6 and α-TNF [42,43]. Additionally, simvastatin rapidly showed favourable effects on the vascular redox state and decreased vascular reactive oxygen species, leading to improved anti-oxidant enzyme status [44,45]. Statins exert beyond cholesterol lowering effect. Indeed, statin involves in restoring or improving endothelial function by reducing oxidative stress and inhibiting inflammatory responses [46].

In this study, high cholesterol diet-fed rat was used as a model to investigate the effectiveness of DPO as a new source of healthy vegetable oil used as a healthy alternative to prevent lipid disturbance. Administration with DPO significantly reduced TC, TG and HMG-CoA-r levels compared with hypercholesterolemic rats (*p* < 0.05). The effects of DPO in lowering TC, TG, and LDL are consistent with that study by Shakirin et al. [4]. The DPO is rich in UFA (53.49 ± 1.89%), which comprises 40.38 ± 0.79% of MUFA and 13.11 ± 1.10% of PUFA. The main component of MUFA in DPO was oleic acid (39.37 ± 1.01%). While among the PUFA, linoleic (cis) acid (omega 6) (12.54 ± 1.03%) and α-linolenic acid (omega 3) (0.44 ± 0.06%) are the most abundant (Table 3).

Previous studies demonstrated that UFA effectively lowered TC, TG, and LDL [47,48,49,50]. Additionally, consumption of both MUFA and PUFA may produce a strong hypocholesterolemic effect. The mechanism by which PUFA alters serum cholesterol is related to its role in limiting protein expression by upregulating mRNA levels and increasing the number of cellular LDL-receptors. The increase in LDL-receptors occurs in hepatocytes, which resulted in a cholesterol influx increase. Moreover, PUFA also decreases de novo lipogenesis and very-low-density lipoprotein (VLDL) secretion by fatty acid synthesis suppression [51].

Meanwhile, the mechanism by which MUFA decreases serum cholesterol levels is less exact. However, MUFA was demonstrated to decrease apolipoprotein C-III mRNA levels, a protein present on LDL particles and a precursor of VLDL. By downregulating this protein, VLDL and LDL concentration are reduced in the circulation [52,53]. Additionally, omega-3 fatty acids are involved in the inhibition of the endogenous synthesis and esterification of cholesterol, which leads to an increase in cholesterol excretion in the bile, and an increase in the bile acid synthesis [54].

On the other hand, rats fed with DPO had the lowest TG. In this study, the LPL activity paralleled that of TG. According to Arunima et al. [50], the increase in this enzyme activity indicates increased TG clearance from the circulation. Feeding rats with DPO shows increased LPL level, and is directly correlated with the lower level of TG. LPL is a triacylglycerol hydrolase, whose activity was significantly increased following DPO supplementation.

Oleic acid is classically known as a heart-healthy substance known for its insignificant risk of cardiovascular disease. Compared with SFA, oleic acid causes greater chylomicron’s secretion and contains higher amounts of TG. Chylomicron carries exogenous dietary TG, whereas VLDL carries TG from the liver [55]. The previous study showed that the postprandial rise in TG returns to the baseline level faster with a high MUFA (oleic acid) test meal compared with a meal containing high saturated fat [56]. The increased clearance of TG from the blood following oleic acid consumption is likely due to the formation of larger sized chylomicrons compared to smaller “VLDL-sized” chylomicrons from saturated fat. Hence, consuming a diet containing MUFA may reduce the risk of small chylomicron penetrating the endothelium, which then become stuck under the endothelium, oxidise and can lead to atherosclerosis [57].

Supplementation with DPO leads to a significant increase of anti-oxidant capacity (TAS, SOD, GPX, CAT) when compared with hypercholesterolemic rats (*p* < 0.05). Moreover, the inflammatory markers (CRP, IL-6. and α-TNF) were significantly reduced following supplementation of DPO when compared with hypercholesterolemic rats (*p* < 0.05). In addition, rats fed with DPO had the lowest MDA level.

Endogenous and exogenous free radicals can cause damage to lipids, proteins, carbohydrates, and nucleic acids by interacting with them and, consequently, generating new free radicals [58]. Oleic acid was demonstrated to show a protective effect against inflammation [59,60]. Meanwhile, omega-3 fatty acids were demonstrated to reduce the oxidative stress through its effect by elevating the activity of anti-oxidant enzymes (CAT and SOD) and reducing the MDA level and ameliorating the hyperlipidemia-induced change in the lipid profile of rats [61].

The intake of omega-6 fatty acids such as linoleic or arachidonic acid leads to decreased CVD via their beneficial effects on inflammatory markers [62]. Both omega-6 and omega-3 fatty acids were shown to have anti-inflammatory properties that can suppress the atherogenic activation of vascular endothelial cells [59]. Additionally, Pischon et al. [63] demonstrated that the combination of omega-6 and omega-3 fatty acids is associated with the lowest inflammation levels (CRP, IL-6, and soluble TNF receptor).

Among all biomolecules, lipids are the most sensitive molecules to free radicals. The double bonds in fatty acids form peroxide products by reacting with free radicals, and lipid radicals can be formed upon removing the electron [64]. Lipid peroxidation represents oxidation of polyunsaturated fatty acids in cell membrane lipids or lipoproteins, besides oxidation in vegetables and food oils rich in PUFA omega-3. The free radical activity can be estimated by measuring the malondialdehyde (MDA) concentration in serum [65].

In this study, corn oil was used in the diet for hypercholesterolemic rats. The commercialised corn oil (brand: Krystal) contained high PUFA (57 g/100 g), while the MUFA and SFA were 29 g/100 g and 14 g/100 g, respectively. Hypercholesterolemic rats showed a significantly elevated level of MDA compared to rats treated with DPO. PUFA structure is sensitive to the lipid peroxidation. This reaction is caused by the action of oxygen-free radicals that attack and degrade PUFAs, which leads to the presence of certain toxic and highly reactive molecules (peroxide products) [66]. Saturated and monounsaturated-rich oils demonstrated higher stability compared to polyunsaturated oils [67].

The fatty acid composition of DPO having near the equal percentage of SFA and MUFA at about 40% each may account for low lipid peroxide content in rats treated with DPO. Given our finding, DPO which is rich in oleic acid, omega-6 and omega-3 effectively elevates anti-oxidant enzymes’ activity and further manages inflammatory markers and control of MDA.

## 4. Conclusions

The MVC, FFA, IV, and PV in DPO showed satisfactory initial quality parameters. Moreover, this study provides evidence that DPO ameliorates hypercholesterolemia by reducing TC, TG, MDA and HMG-CoA-r levels. Improvement in LPL, anti-oxidant defences (TAS, SOD, GPx, and CAT) and inflammatory markers (CRP, IL-6. and α-TNF) were also observed in the rats fed with DPO. The beneficial effects of DPO on hypercholesterolemia seem to be attributed to its balanced fatty acid composition’s synergistic effects. The results on the quality and efficacy of locally made SC-CO_2_ extracted DPO suggest that the DPO could be used as a healthy alternative fat source. Further investigation of DPO for culinary applications warrants its potential for commercialisation.

## Figures and Tables

**Table 1:** Experimental diets.

Ingredients (g)	Diets		
	ND	HC	HC + 2% DPO
Corn starch	180	170	170
Sucrose	500	500	500
Casein	120	120	120
Vitamin mixture	10	10	10
Mineral mixture	35	35	35
Cellulose	50	50	50
DL-methionine	3	3	3
Choline	2	2	2
Ghee	80	80	80
Corn oil	20	20	-
Cholesterol	-	10	10
Dabai pulp oil	-	-	20
Total	1000	1000	1000
^1^ Energy (kcal/100 g)	397	423	387

ND, normal diet; HC, high cholesterol diet; DPO, dabai pulp oil. ^1^ Energy (kcal/100 g) represents the calories content in 100 g of diet.

**Table 2:** Quality parameters of DPO and palm oil.

Quality Parameters	DPO	Palm Oil ^1^
Moisture and volatile content (%)	<0.001 ± 0.00	0.25
Free fatty acid content (%, as in oleic acid)	2.57 ± 0.03	5.0
Iodine value (g iodine/100 g oil)	53.74 ± 0.08	50.1–54.9
Peroxide value (mEq/kg)	4.97 ± 0.00	2.0

^1^ Specification value for crude palm oil as specified by Malaysian Standard MS814:2007.

**Table 3:** Fatty acid composition of DPO.

Fatty Acids		% in Fat ^1^
Saturated fatty acids		
C8	Caprylic	0.05 ± 0.00
C10	Capric	0.01 ± 0.00
C11	Undecanoic	0.01 ± 0.01
C12	Lauric	0.74 ± 0.06
C14	Myristic	0.28 ± 0.00
C15	Pentadecanoic	0.03 ± 0.00
C16	Palmitic	41.56 ± 0.10
C17	Heptadecanoic	0.11 ± 0.00
C18	Stearic	4.31 ± 0.01
C20	Arachidic	0.10 ± 0.00
C21	Heneicosanoic	0.02 ± 0.01
C22	Behenic	0.23 ± 0.06
C23	Tricosanoic	0.11 ± 0.00
C24	Lignoceric	0.10 ± 0.01
Monounsaturated fatty acids		
C14:1	Myristoleic	0.03 ± 0.02
C15:1	Cis-10-pentadecenoic	0.04 ± 0.01
C16:1	Palmitoleic	0.63 ± 0.02
C17:1	Cis-10-heptadecanoic	0.03 ± 0.00
C18:1n9c	Oleic	39.37 ± 1.01
C20:1n9	Cis-11-eicosenoic	0.07 ± 0.01
C22:1n9	Erucic	0.03 ± 0.03
C24:1	Nervonic	0.20 ± 0.19
Polyunsaturated fatty acids		
C18:2n6c	Linoleic (cis)	12.54 ± 1.03
C18:3n6	ϒ-linolenic	0.12 ± 0.02
C18:3n3	α-linolenic	0.44 ± 0.06
C20:4n6	Arachidonic	0.01 ± 0.01
Saturated fatty acids		47.65 ± 0.11
Monounsaturated fatty acids		40.38 ± 0.79
Polyunsaturated fatty acids		13.11 ± 1.10

^1^ % in fat indicates mean of weight % in total fatty acids. Results are given as mean ± SD (n = 3).

**Table 4:** The beneficial effects of DPO treatment diet on hypercholesterolemic rats.

Group	NG	PG	HG	SG
TC (mmol/L)	1.57 ± 0.15 ^a^	2.12 ± 0.65 ^b^	1.51 ± 0.18 ^a^	1.23 ± 0.05 ^a^
TG (mmol/L)	1.97 ± 0.92	2.08 ± 0.65	1.10 ± 0.30 ^ab^	1.49 ± 0.28
LDL (mmol/L)	0.17 ± 0.06 ^a^	0.50 ± 0.19 ^b^	0.42 ± 0.13 ^b^	0.24 ± 0.05 ^a^
HDL (mmol/L)	1.36 ± 0.14	1.27 ± 0.53	1.22 ± 0.16	1.08 ± 0.07 ^b^
HMG-CoA-r (ng/mL)	1.47 ± 0.07 ^a^	2.02 ± 0.24 ^b^	1.64 ± 0.12 ^ab^	1.39 ± 0.04 ^a^
LPL (ng/mL)	11.77± 1.37	12.34 ± 1.43	15.45 ± 2.09 ^ab^	13.64 ± 1.62
TAS (U/mL)	2.24 ± 0.31 ^a^	1.95 ± 0.22 ^b^	2.34 ± 0.22 ^a^	1.91 ± 0.25 ^b^
SOD (ng/mL)	0.78 ± 0.13 ^a^	0.58 ± 0.02 ^b^	0.83 ± 0.09 ^a^	0.73 ± 0.17 ^a^
GPx (U/L)	295.98 ± 3.40 ^a^	282.72 ± 14.98 ^b^	301.24 ± 12.86 ^a^	293.51 ± 5.83
CAT (U/mL)	12.96 ± 1.19 ^a^	4.26 ± 0.69 ^b^	17.86 ± 5.09 ^ab^	11.34 ± 1.37 ^a^
MDA (ng/mL)	142.88 ± 37.94 ^a^	206.82 ± 51.52 ^b^	125.71 ± 16.52 ^a^	149.48 ± 32.94 ^a^
CRP (ng/mL)	0.85 ± 0.16 ^a^	1.07 ± 0.20 ^b^	0.75 ± 0.15 ^a^	0.76 ± 0.28 ^a^
IL-6 (pg/mL)	315.32 ± 31.28 ^a^	364.97 ± 49.84 ^b^	252.02 ± 15.19 ^ab^	255.56 ± 21.56 ^ab^
α-TNF (pg/mL)	221.67± 14.36 ^a^	293.76 ± 20.41 ^b^	191.88 ± 6.49 ^ab^	191.88 ± 8.63 ^ab^

NG, normal rats group; PG, hypercholesterolemic positive control group; HG, hypercholesterolemic rats treated with 2% DPO group; SG, hypercholesterolemic rats simvastatin treated group; TC, total cholesterol; TG, triglyceride; LDL, low-density lipoprotein; HDL, high-density lipoprotein; HMG-CoA-r, 3-hydroxy-3-methylglutaryl-CoA reductase; LPL, lipoprotein lipase; TAS, total anti-oxidant status; SOD, superoxide dismutase; GPx, glutathione peroxide; CAT, catalase; MDA, lipid peroxidation, CRP, C-reactive protein; IL-6, interleukin 6; α-TNF, tumour necrosis factor-α. ^a^ Indicates a statistically significant difference (*p* < 0.05) versus the PG group; ^b^ Indicates a statistically significant difference (*p* < 0.05) versus the NG group by Duncan’s multiple range tests using SPSS for Windows version 23. Results are given as mean ± SD (n = 6).

## Data Availability

The data presented in this study are available on request from the corresponding author.

## Abbreviation

α-TNF: tumour necrosis factor-α; CAT, catalase; CM, chloroform-methanol; CRP, C-reactive protein DPO, dabai pulp oil; FFA, free fatty acid; GPx, glutathione peroxide; HDL, high-density lipoprotein; HG, hypercholesterolemic rats treated with 2% DPO group; HMG-CoA-r, 3-hydroxy-3-methylglutaryl-CoA reductase; IV, iodine value; IL-6, interleukin 6; LDL, low-density lipoprotein; LPL, lipoprotein lipase; MDA, lipid peroxidation; MUFA, monounsaturated fatty acid; MVC, moisture and volatile content; NG, normal rats group; PE, petroleum-ether; PG, hypercholesterolemic positive control group; PUFA, polyunsaturated fatty acid; PV, peroxide value; SC-CO_2_, supercritical fluid extraction; SFA, saturated fatty acid; SFE, supercritical fluid extraction; SG, hypercholesterolemic rats simvastatin treated group; SOD, superoxide dismutase; TAS, total anti-oxidant status; TC, total cholesterol; TG, triglyceride; UFA, unsaturated fatty acid; VLDL, very-low-density lipoprotein.

## Appendix A

Preparation of Fatty Acid methyl esters (FAMEs) was performed based on the IUPAC 2.301 protocol [68]. Appendix A Table A1 shows the Gas chromatography (GC) for FAMES analysis. The fatty acid content in DPO was quantified and determined based on the FAMEs (Supelco 37 Component FAME Mix, Cat. No: 18919, Sigma Aldrich, MO, USA).

**Table A1:** Gas chromatography conditions for fatty acid methyl esters (FAMEs) analysis.

Description	Condition
Brand	HP 5890 series II plus
Split/Column injector	Split/splitless
Column	DB 225 (30 m × 0.25 mm × 0.25 mm)
Carrier gas and flow rate	H_2_, 1.3 mL/min
Oven temperature	30–250 °C
Detector temperature	240 °C
Make-up gas	N_2_
Inlet pressure	20 psi, 1 mL/min
Overall run time	30 min
Software	Chemstation
